# Specific lncRNA signatures discriminate childhood acute leukaemias: a pilot study

**DOI:** 10.1186/s12935-022-02789-3

**Published:** 2022-11-30

**Authors:** Lorena Buono, Concetta Iside, Antonia De Matteo, Pio Stellato, Giuliana Beneduce, Roberta Penta de Vera d’Aragona, Rosanna Parasole, Marco Salvatore, Giovanni Smaldone, Peppino Mirabelli

**Affiliations:** 1 IRCCS SYNLAB SDN, Via E. Gianturco 113, 80413 Naples, Italy; 2 Santobono-Pausilipon Children’s Hospital, AORN, Naples, Italy

**Keywords:** T-ALL, B-ALL, lncRNA, Transcriptome, Leukaemia, Biomarkers

## Abstract

**Background:**

Long non-coding RNAs are RNAs longer than 200 bps that do not encode any proteins and are able to alter gene expression by acting on different steps of regulation, including DNA methylation and chromatin structure. They represent a class of biomarkers of crescent interest in the hematologic and oncologic fields. Recent studies showed that the expression levels of specific lncRNAs correlate with the prognosis of paediatric patients with Acute Lymphoblastic Leukaemia.

**Methods:**

We used NGS approaches to analyse the transcriptome of 9 childhood B-ALL patients and 6 childhood T-ALL patients, in comparison with B and T healthy lymphocytes from cord blood. We validate our findings both ex vivo, in a different cohort of 10 B-ALL and 10 T-ALL patients, and in silico using public datasets.

**Results:**

We characterised the lncRNA landscape for B-ALL, T-ALL, healthy B, and T cell progenitors. From the characterised signature, we selected candidate lncRNAs able to discriminate not only B-ALL and T-ALL from healthy subjects but also between the two types of leukaemia, and subsequently validated their potential as a diagnostic tool in an additional cohort of paediatric patients. We confirmed our finding with open access transcriptomic data, comparing ALL lncRNAs with AML lncRNA landscape as well. Finally, expression correlation analyses of T-ALL selected lncRNA biomarkers suggested a possible role in lymphocyte activation and the β-catenin signalling pathway for AC247036.1 and involvement in *hedgehog* signalling for HHIP-AS1.

**Conclusions:**

Our work identified a lncRNA signature discriminating paediatric B-ALL and T-ALL from healthy subjects, between them and from AML. This study provides the keystone to future clinical studies determining the theragnostic value of the characterised long non coding transcriptome panorama in a clinical setting for childhood patient management.

**Supplementary Information:**

The online version contains supplementary material available at 10.1186/s12935-022-02789-3.

## Background

Leukaemia, a cancer of the blood cells, is the most common type of cancer in children and adolescents. The leukemic cells arise from the abnormal and clonal proliferation of hematopoietic progenitor cells, leading to disruption of normal marrow function and determining haematopoiesis failure. In addition, the leukemic cells rapidly move through the bloodstream and crowd out healthy blood cells, increasing the body's chances of infection and other complications. There are two main subtypes of paediatric acute leukaemia: the commoner acute lymphoblastic leukaemia (ALL) and the rarer acute myeloid leukaemia (AML) [1]. ALL arises from the malignant transformation and aberrant proliferation of B cell progenitors (about 85% of cases) or T cell progenitors (about 15% of cases). B-cell acute lymphoblastic leukaemia (B-ALL) is the most common form of ALL, associated with distinct gene expression profiles and driven by three main types of initiating genetic alteration: (i) chromosomal aneuploidy; (ii) rearrangements that deregulate oncogenes or encode chimeric transcription factors, and (iii) point mutations [2]. T-cell acute lymphoblastic leukaemia (T-ALL) is less frequent than B-ALL and has a worse prognosis. Indeed, although current chemotherapy protocols and stem cell transplantation have achieved good results, T-ALL paediatric patients have a poor prognosis: about 20–30% of patients relapse, with a 5-year survival of approximately 20% for T-ALL patients [3, 4]. Childhood T-ALL is featured by recurrent alterations mostly deregulating three pathways: (i) expression of T-lineage transcription factors, (ii) NOTCH1/MYC signalling, and (iii) cell-cycle control [5, 6]. The etiopathogenic mechanisms leading to leukemic transformation are still largely unknown, but genetic, immunologic, viral, and environmental factors have been implicated [7–9]. Today, the classification of ALL into risk groups is based on the assessment of minimal residual disease assessed by molecular biology and cytometry during treatment combined with the analysis of poor prognosis genetic aberrations (e.g., t(4;11), t(17;19) etc.) [10].

Childhood AML is a more heterogeneous disease associated with poor outcomes. It is characterised by immature clonal myeloid cells’ proliferation and aberrant differentiation [11]. This hematologic malignancy encloses a wide spectrum of genomic insults and molecular alterations that influence clinical outcomes and provide potential targets for personalised therapy [12]. In ALL and AML, the classification into risk groups is the first and crucial step towards tailored patient management and facilitates a targeted approach with the most appropriate therapeutic treatment. The last decade has witnessed great advances in our understanding of the genetic and biological basis of childhood acute leukaemia, the improvement of experimental models to probe mechanisms and evaluate new therapies, and the development of more efficacious treatment stratification such as the recently introduced molecularly targeted therapy and immunotherapy [13, 14]. The onset of high-throughput sequencing and bioinformatic approaches have revolutionised our understanding of the molecular taxonomy of childhood leukaemia [15]. These modern applications of next-generation sequencing (NGS) technology have uncovered considerable heterogeneity and molecular complexity within this paediatric haematological disease, based on the interplay of genomic mutations, epigenetic remodelling, transcriptome misregulation, and aberrant cell signalling and proliferation pathways [16]. Many of these alterations may have important implications for the diagnosis and risk-stratification, highlighting the importance of implementing genome and transcriptome characterization in the clinical management of acute leukaemia to facilitate more accurate risk-stratification and, in some cases, targeted therapy.

The recent transcriptome-wide gene expression studies not only characterised the mRNA misregulation of ALL resulting from aberrant functioning of transcription factors, epigenetic rearrangements, structural variants, or chromosome mutations [17], but they have also uncovered evidence of significant relationships between lncRNAs dysregulation and malignant hematopoietic transformation, with specific lncRNAs gaining interest as diagnostic biomarkers, novel therapeutic targets, and predictors of clinical outcomes [18, 19]. LncRNAs are transcripts usually longer than 200 bp and lacking an open reading frame. They can alter gene expression by acting on different steps of regulation, including chromatin modification, transcription, splicing, RNA transport, and translation [20, 21]. However, the precise role that lncRNA expression plays in the pathogenesis of paediatric ALL has been scarcely studied and even less understood.

Here we want to present the transcriptome-wide analysis of polyadenylated long non-coding RNA profiles in B-ALL and T-ALL cases matched with a control population composed of normal cord blood-derived T cells and B-cells. A specific lncRNA signature was identified to distinguish leukemic B- and T-ALL, normal lymphoid B and T cells, and AML.

## Methods

### Study population

The procedures followed in the present study are in line with the Helsinki declaration and have been approved by the local ethical committees of the IRCCS-SDN (Ethical Committee IRCCS Pascale, Naples, Italy—protocol number 5/19 of the 19/06/2019) and the AORN Santobono-Pausilipon (Ethical Committee Cardarelli/Pausilion, Naples Italy—protocol number 07/20 of 03/06/2020). Both parents signed informed consent and all participants provided informed assents. All children enrolled in the study were included at moment of diagnosis, patients’ clinical features are presented in Tables 1 and 2 and Additional file 1: Dataset S1 (B-ALL patients) and in Tables 3 and 4 and Additional file 2: Dataset S2 (T-ALL patients).

**Table 1:** Clinical information of B-ALL patients used for RNA-seq experiment

B-ALL Patients for RNA-seq experiment			
Sex	Age (years)	Race	WBC diagnosis (Blasts/mmc)
F	12	Caucasian	1750
F	6	Caucasian	15,200
F	4	Caucasian	29,820
F	7	Caucasian	518,000
F	14	Caucasian	16,570
F	3	Caucasian	5960
M	15	Caucasian	192,900
M	17	Caucasian	131,000
M	5	Caucasian	4500

*WBC* white blood count, *mmc* cubic millimetres

**Table 2:** Clinical information of B-ALL patients used for validation experiments

B-ALL Patients for RT-PCR validation			
Sex	Age (years)	Race	WBC diagnosis (Blasts/mmc)
M	5	Caucasian	1260
F	6	Caucasian	1370
F	3	Caucasian	5160
M	2	Caucasian	18,650
M	3	Caucasian	67,020
M	17	Caucasian	131,000
M	5	Caucasian	4500
M	10	Caucasian	31,520
M	3	Caucasian	11,940
F	4	Caucasian	1700

*WBC* white blood count, *mmc* cubic millimetres

**Table 3:** Clinical information of T-ALL patients used for RNA-seq experiment

T-ALL Patients for RNA seq experiment			
Sex	Age (years)	Race	WBC Diagnosis (Blasts/mmc)
M	8	Caucasian	368,120
F	6	Caucasian	212,850
M	2	Caucasian	500,000
M	8	Caucasian	447,000
M	17	Asiatic	303,440
F	4	Syrian	583,000

*WBC* white blood count, *mmc* cubic millimetres

**Table 4:** Clinical information of T-ALL patients used for validation experiment

T-ALL Patients for RT-PCR validation			
Sex	Age (years)	Race	WBC diagnosis (Blasts/mmc)
F	16	Caucasian	1960
M	9	Caucasian	6280
M	2	Caucasian	500,000
M	7	Maroccan	52,840
M	16	Caucasian	120,700
M	0	Caucasian	177,000
M	10	Caucasian	447,000
M	13	Caucasian	30,970
M	9	Caucasian	16,280
F	11	Caucasian	262,080

*WBC* white blood count, *mmc* cubic millimetres

### RNA sequencing

Total RNA was extracted from leukemic cells derived from Bone Marrow blood of B-ALL and T-ALL patients and purified B lymphocytes and T lymphocytes from cord blood of healthy donor using Trizol (Thermo Fischer Scientific, Waltham, MA, USA) reagent protocol, according to manufacturer instructions. RNA concentration and quality were determined using Qubit (ThermoFisher Scientific, MA, USA) spectrophotometer. RNA-seq libraries were prepared with 3′-DGE approach and sequenced SEx100 on an Illumina Novaseq platform.

### Bioinformatic analysis

FASTQ files were aligned with STAR v. 2.7.1a [22] on the GRCh38 human genome. Raw counts were obtained using HTSeq v. 2.0.0 [23]. Normalisation and differential expression analysis were performed with DESeq2 v. 1.36.0 [24]. LncRNA annotations were done with Biomart v. 2.52.0 [25]. Hierarchical clustering and heatmap representations were performed as in Buono et al. [26]. Functional enrichment analysis of lncRNA clusters were analysed with gProfiler [27]). Expression correlations were calculated with “pearson” method in R. Statistical significance of gene overlapping were calculated using Fisher’s exact test through the R package GeneOverlap v. 0.99.0. GO enrichment analyses for correlated genes were performed with EnrichR [28] using as input the list of all the positively correlated genes with p-value < 0.001.

### Real time PCR analyses

Total RNA was extracted from B-ALL, T-ALL and PBMCs derived from cord blood using the Trizol Reagent protocol. After extraction, RNA was quantified using NanoPhotometer NP80 (Implen, USA). Next, 1 µg of total RNA from each sample was reverted in cDNA using SuperScript III First-Strand Synthesis SuperMix kit (Thermo Fisher Scientific) according to the manufacturer’s protocol. The expression level of selected lncRNAs was measured by qRT-PCR using the following formula: 2-∆Ct on C1000 Touch Thermal Cycler (Bio-Rad, Hercules, CA, USA) using iQ SYBR Green Supermix (#1708882, Bio-Rad). Ribosomal Protein S18 (RPS18) level was used as an endogenous control to normalize lncRNAs expression. The following primers were used:

RPS18: fw 5′ - CGATGGGCGGCGGAAAATA-3′; rev 5′—CTGCTTTCCTCAACACCACA-3′

LINC00958: fw 5′ -TGCAGCAAGATAGCTCCAGG-3′; rev 5′- CCTGGCGTCTGTGTAGTGTT-3′

LINC00114: fw 5′- TAGAGGCCTGATGGAGTGGA-3′; rev 5′- CTGCCCAGGAAACTGTAGGT-3′

AL713998: fw 5′- AACATTTGGTGCCGAAAGCC-3′; rev 5′- GCGAGGGAAGTCTCTTGCAT-3′

AC008060: fw 5′- CGAGGCTTGGACAAATGCAG-3′; rev 5′- CAGTCCCAAAGGAAGCGGAT-3′

AL590226: fw 5′- GAATCCACAGATGGCGTGTG-3′; rev 5′- TCAGGTAGCTGCGAGTTCAA-3′

PCAT18: fw 5′- GTC CCA GCA CTT CAC TGG TT-3′; rev 5′- AGC TGG GAT ATG GTA GCA GC-3′

HHIP-AS1: fw 5′-TCA CAC CAC CAC TGA GCA AC-3′; rev 5′- AGC TCT GCT TGG TGA ATG GA-3′

AC247036: fw 5′- TGT CCT GTG GTG GGA AAA ACA-3′; rev 5′- ACC CGG GAG TCA TCT GAA CA-3′

LINC01222: fw 5′- AGCAGGGGTAACATTATGGGC-3′; rev 5′-AGC TGC TCC CCC TTT ATC TTC-3′

AC116351.1: fw 5′- TGGAAAGTCCAGCGACAGAC-3′; rev 5′: GTCTCCCTTCACAGTGGCAA - 3′

## Results

### Long non-coding RNA landscape in childhood acute lymphoblastic leukaemia

**Fig. 1:**
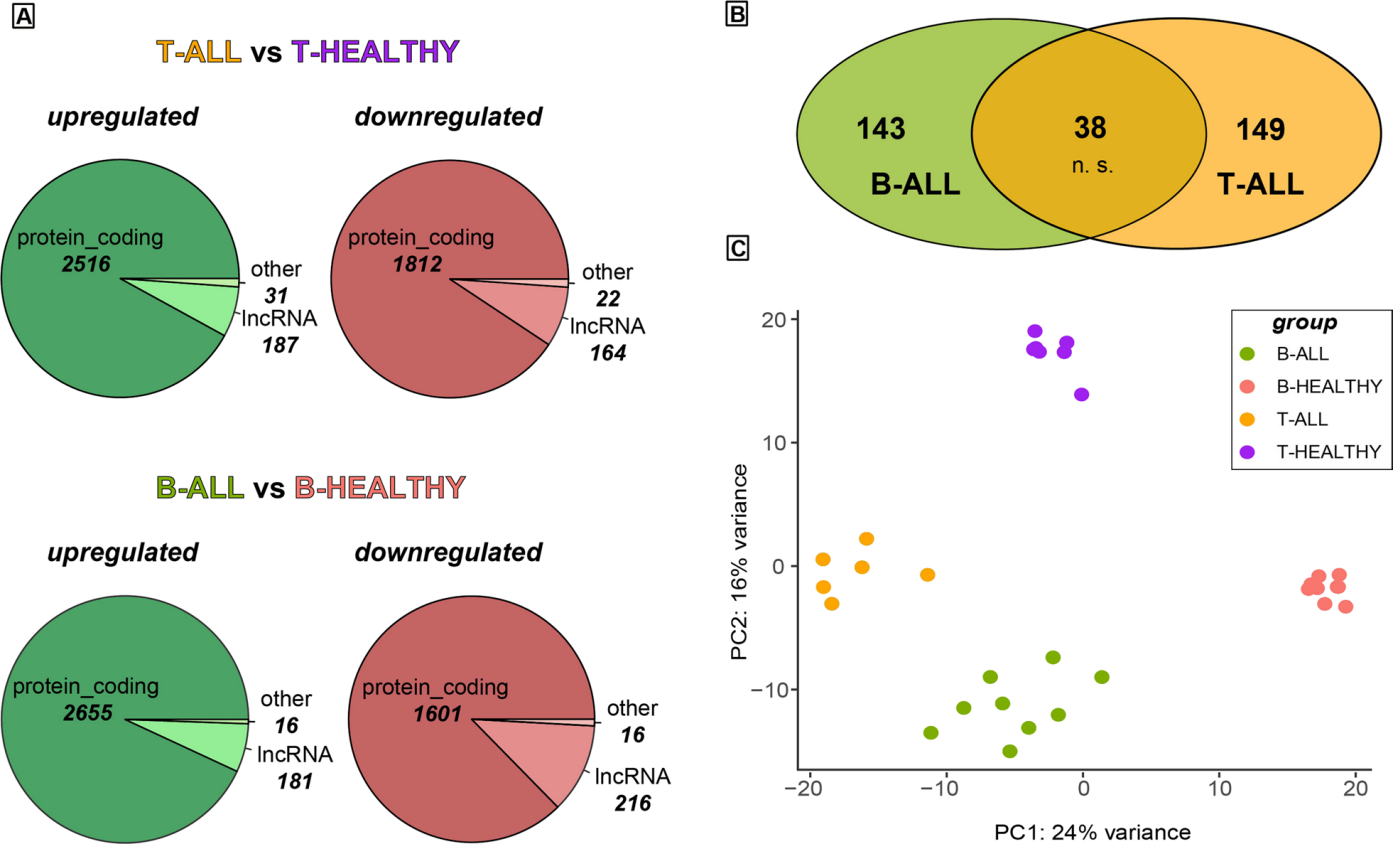
Transcriptomic landscape of childhood ALL. **A** Pie chart showing the number of significantly (adjusted p-value < 0.05) upregulated and downregulated genes between T-ALL and T naïve cells (upper panel) and B-ALL and B naïve cells (lower panel). The charts indicate the number of protein coding and lncRNAs differentially regulated. **B** Number of common lncRNAs between the lncRNAs upregulated in T-ALL vs T naïve cells and the lncRNAs upregulated in B-ALL vs B naïve cells. Fisher’s exact test confirmed the overlapping to be not significant with a p-value = 0.088. **C** PCA of the global lncRNA panorama of all the samples included in differential expression analysis

To gain insight into the transcriptomic landscape of childhood acute lymphoblastic leukaemia, we performed RNA-seq on RNA samples from the bone marrow of paediatric patients. We used purified naïve lymphocytes from cord blood as healthy control. In the case of acute T-cell lymphoblastic leukaemia (T-ALL), we sequenced 6 childhood T-ALL samples and 7 purified naïve T cells from cord blood (T-ALL patient clinical details in Additional file 2: Dataset S2). For acute B-cell lymphoblastic leukaemia (B-ALL), we used 9 samples from patients and 9 purified naïve B cells from cord blood (B-ALL patient clinical details in Additional file 1: Dataset S1). Differential expression analysis between leukaemia patients and the relative healthy controls revealed a comparable number of significant upregulated and downregulated genes (adjusted p-value < 0.05) in the two subtypes of acute lymphoblastic leukaemia. In T-ALL, we observed 2734 upregulated and 1998 downregulated genes (complete T-ALL differential expression analysis can be found in Additional file 3: Dataset S3). These differentially regulated genes include 187 upregulated and 164 downregulated lncRNAs. From the comparison between B-ALL patients and the healthy controls, we obtained 2852 upregulated genes and 1833 down-regulated ones (complete B-ALL differentially expression analysis can be found in Additional file 4: Dataset S4), including 181 and 216 lncRNAs, respectively (Fig. 1A). When comparing the landscape of upregulated lncRNAs in B-ALL and T-ALL, we noticed a very poor signature overlapping, with only 38 lncRNAs in common, whereas 143 and 149 are specific for the B-ALL and T-ALL disease, respectively (Fig. 1B). The overlapping of 38 lncRNAs resulted to be not significant, while the same analysis for the whole transcriptome or only for the protein coding genes showed a significant greater proportion of overlapping genes (Additional file 11: Fig. S1A and B). This data points to a sharper specificity of lncRNA core underlying oncogenic events in each type of childhood acute leukaemia. Based on this result, we performed a principal component analysis (PCA) of the whole lncRNA landscape in the four conditions examined. The graphical representation of the analysis shows a clear separation of the four groups in distinct clusters, indicating the expression of a unique lncRNA signature characterising each condition (Fig. 1C). PCA of the whole transcriptome and protein coding genes scored a slightly wider separation (Additional file 11: Fig. S1C and D), but this can be attributed to the larger number of genes taken in account for clustering, since lncRNAs represent a minority portion of the transcriptomic landscape compared to protein coding genes.

### Identification of long non-coding RNA signature distinguishing ALL disease

**Fig. 2:**
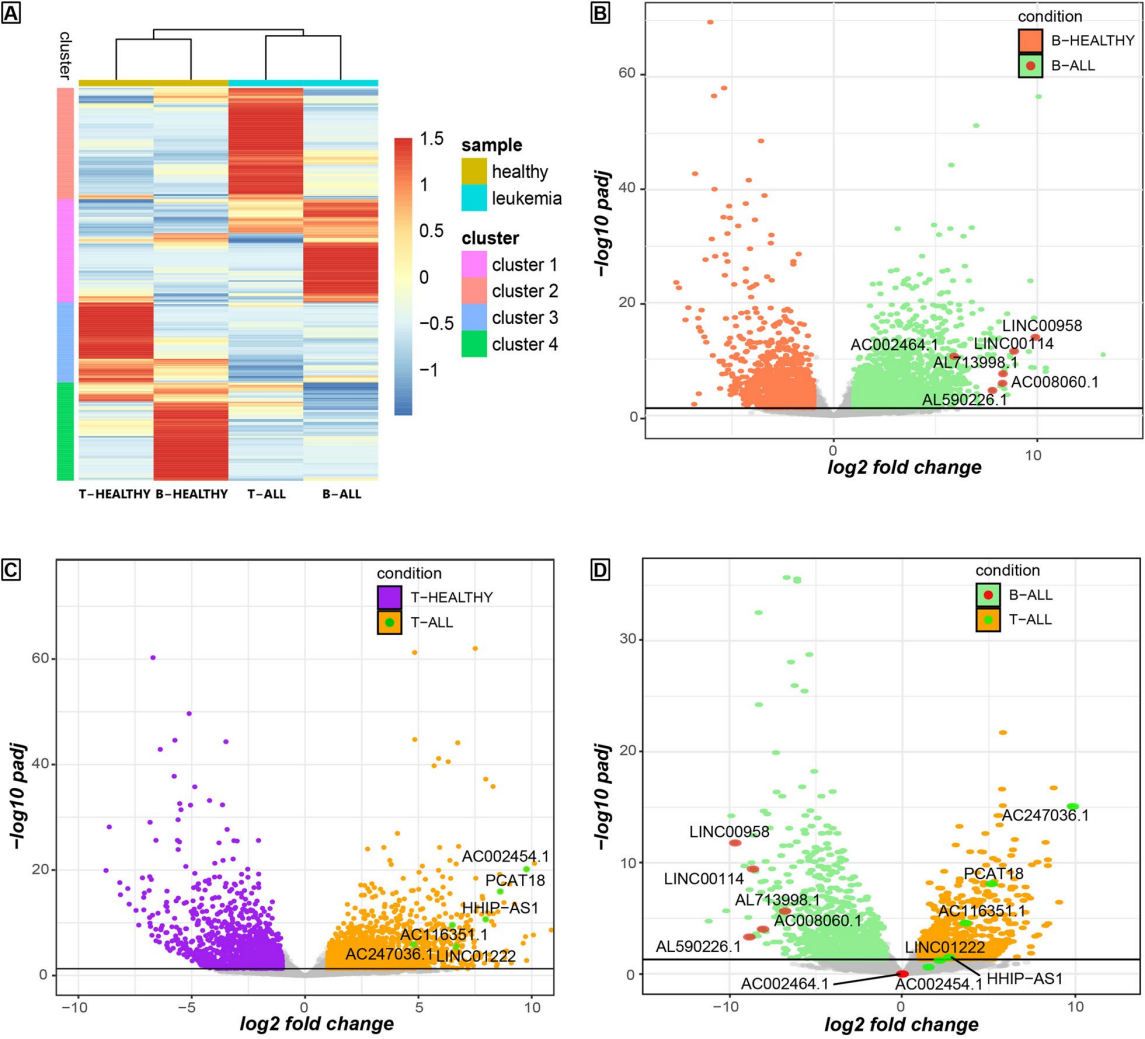
LncRNA signature in childhood ALL. **A** Hierarchical clustering output shows lncRNA expression trends in the distinct conditions. Expression values, normalized by row, are indicated with a red (highest expression) to blue (lowest) graded colour. Each cluster comprise the lncRNA signature of a particular condition, one ALL subtype or the healthy counterparts. **B** Volcano plot illustrating the transcriptome variations between B-ALL and B naïve cells. Each dot corresponds to a gene. Grey dots indicate not significant variations (adjusted p-value > 0.05) and significant variations with a log2(fold change) between 1 and − 1. Red dots highlight the position of interesting lncRNAs reported with text in the figure. **C** Volcano plot showing the transcriptome variations between T-ALL and T naïve cells. Significance and fold change threshold are reported as in **B**. Green dots highlight the position of interesting lncRNAs reported with text in the figure. **D** Volcano plot displaying the transcriptome variations between B-ALL and T-ALL. Significance and fold change threshold are reported as in **B**. Red dots mark the position of the lncRNAs highlighted in **B**, green dots mark the ones highlighted in **C**

After a general characterization of the transcriptomic landscape of childhood T-ALL and B-ALL, we performed an unsupervised hierarchical clustering analysis of all the lncRNAs significantly differentially expressed in at least one of the following comparisons: T-ALL vs. T-healthy, B-ALL vs. B-healthy or T-ALL vs. B-ALL. This method of selection of the input lncRNAs for the hierarchical clustering analysis was chosen to eliminate all the lncRNAs that are not likely to be neither directly involved in the leukemogenesis of B and T lymphoblasts nor vary between the two types of disease and thus could have generated a high level of background, lowering the sharpness and accuracy of the analysis. This approach identified 4 main expression clusters, which are distinctive for each condition, delineating a specific molecular signature of the disease capable of distinguishing not only between healthy and ALL patients but also between B-ALL and T-ALL patients (Fig. 2A, complete list of lncRNA molecular signature in Additional file 5: Dataset S5). This analysis not only defined the lncRNA signature for each condition but also aggregated the samples in two sub-groups based on similarity, showing a higher resemblance between the two ALL signatures and the two healthy signatures than between the ALL subtype and its respective control. Functional enrichment analysis for the list of lncRNAs belonging to T-ALL cluster (cluster 2) returned no significant enrichment besides expected gene ontology terms such as “regulatory RNA binding” (GO:0061980) or “negative regulation of miRNA-mediated gene silencing” (GO:0035198) (data not shown). This may also be due to the poor characterisation and annotation in dedicated databases of the lncRNAs being investigated. Yet, interestingly the same analysis for the lncRNAs belonging to B-ALL signature (cluster 1) outputted the WikiPathway [29] term “CCL18 signalling pathway” with adjusted p-value = 6.060 × 10^–4^ (data not shown). CCL18 is a chemokine produced mainly by antigen-presenting cells of the innate immune system, such as dendritic cells, monocytes, and macrophages. A study from Catusse et al. suggested that CCL18 might be an important factor interfering with pathophysiological homing and maturation processes of B-ALL cells through the chemokine receptor CXCR4 [30]. Our data corroborate this hypothesis and suggest that this process could be mediated by lncRNAs part of the identified B-ALL signature.

We selected 12 lncRNAs that have never been associated with childhood ALL previously for further validations, 6 from the group of specific lncRNAs for B-ALL (cluster 1: LINC00958, LINC00114, AL713998.1, AC008060.1, AL590226.1, AC002464.1) and 6 from the group of specific lncRNAs for T-ALL (cluster 2: AC002454.1, PCAT18, HHIP-AS1, AC116351.1, AC247036.1, LINC01222). All these lncRNAs were extremely upregulated when comparing B-ALL or T-ALL with their relative healthy control (Fig. 2B and C). From our transcriptomic data, most of these selected lncRNAs can also discriminate between B-ALL and T-ALL; AC002464.1 (B-ALL signature), LINC01222, and AC002454.1 (T-ALL signature) are not differentially expressed between the two subtypes of acute lymphoblastic leukaemia (complete differential analysis B-ALL VS T-ALL can be found in Additional file 6: Dataset S6).

### Expression of long non-coding RNA candidates in B-ALL and T-ALL study cohort

**Fig. 3:**
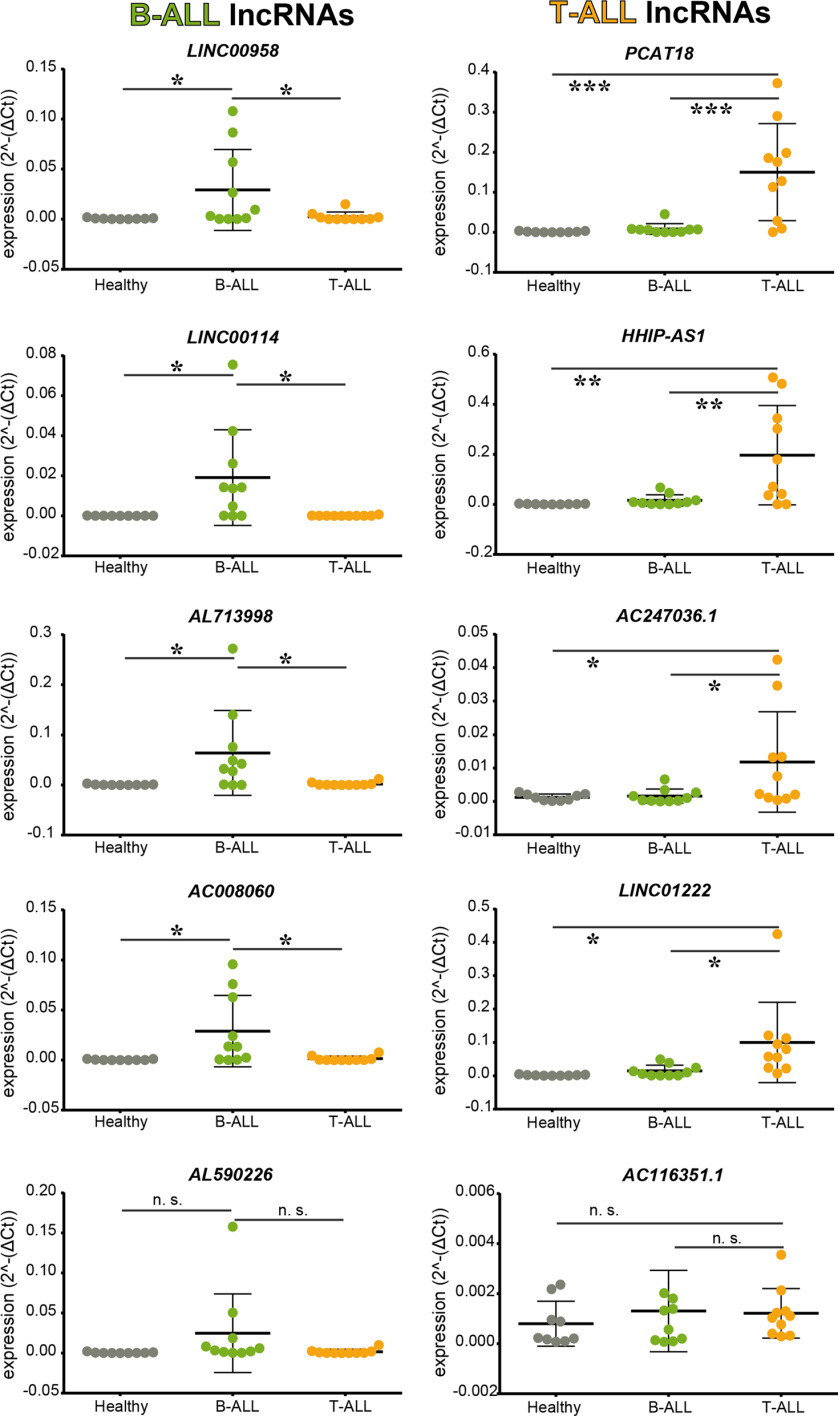
RT-PCR validations on larger cohorts of patients. Expression level of selected lncRNAs in PBMC derived from healthy subjects and leukemic cells derived from paediatric B-ALL and T-ALL patients. Expression levels were plotted according to the relative expression (2-ΔCt method) measured in PBMC from healthy donors (n = 10, grey circles), in bone marrow cells from diagnosed paediatric B-ALL patients (B-ALL, n = 10, green circles) and in paediatric T-ALL patients (T-ALL, n = 10, orange circles). *p < 0.05, **p < 0.01; Mann Whitney t-test

To confirm our omics analyses, we decided to test selected lncRNAs expression levels in a different cohort of 10 B-ALL and 10 T-ALL patients compared to PBMCs derived from cord blood. Figure 3 (left panels) reported that four of five selected lncRNAs (LINC00958, LINC00114, AL713998, and AC008060) were significantly over-expressed in B-ALL patients compared to both healthy and T-ALL samples. No significant differences occur in the expression level of these lncRNAs comparing T-ALL patients and Healthy subjects. Only for one selected lncRNA (AL590226), the bioinformatic analyses were not confirmed since its expression levels were not significantly higher in B-ALL patients with respect to Healthy subjects and T-ALL patients. Just as for de-regulated lncRNAs in B-ALL patients, we also performed validation for deregulated lncRNAs in T-ALL patients using the same study cohort.

As reported in Fig. 3 (right panels), the lncRNA PCAT18, HHIP-AS1, AC247036.1, and LINC01222 results were significantly higher in T-ALL patients with respect to Healthy subjects and B-ALL patients. Also, in this case, for one of the selected lncRNA (AC116351.1) no significant differences were observed through the three populations analysed.

We correlated the expression levels of the analysed lncRNAs with patient clinical information. Interestingly, despite our limited sample size, we found a significant correlation of HHIP-AS1 expression levels with patient age; the higher lncRNA expression, the younger the patients. Further, AC247036.1 expression negative correlates with WBC counts at diagnosis (Additional file 11: Fig. S2).

### LncRNA signature as a molecular biomarker of different types of acute childhood leukaemia

**Fig. 4:**
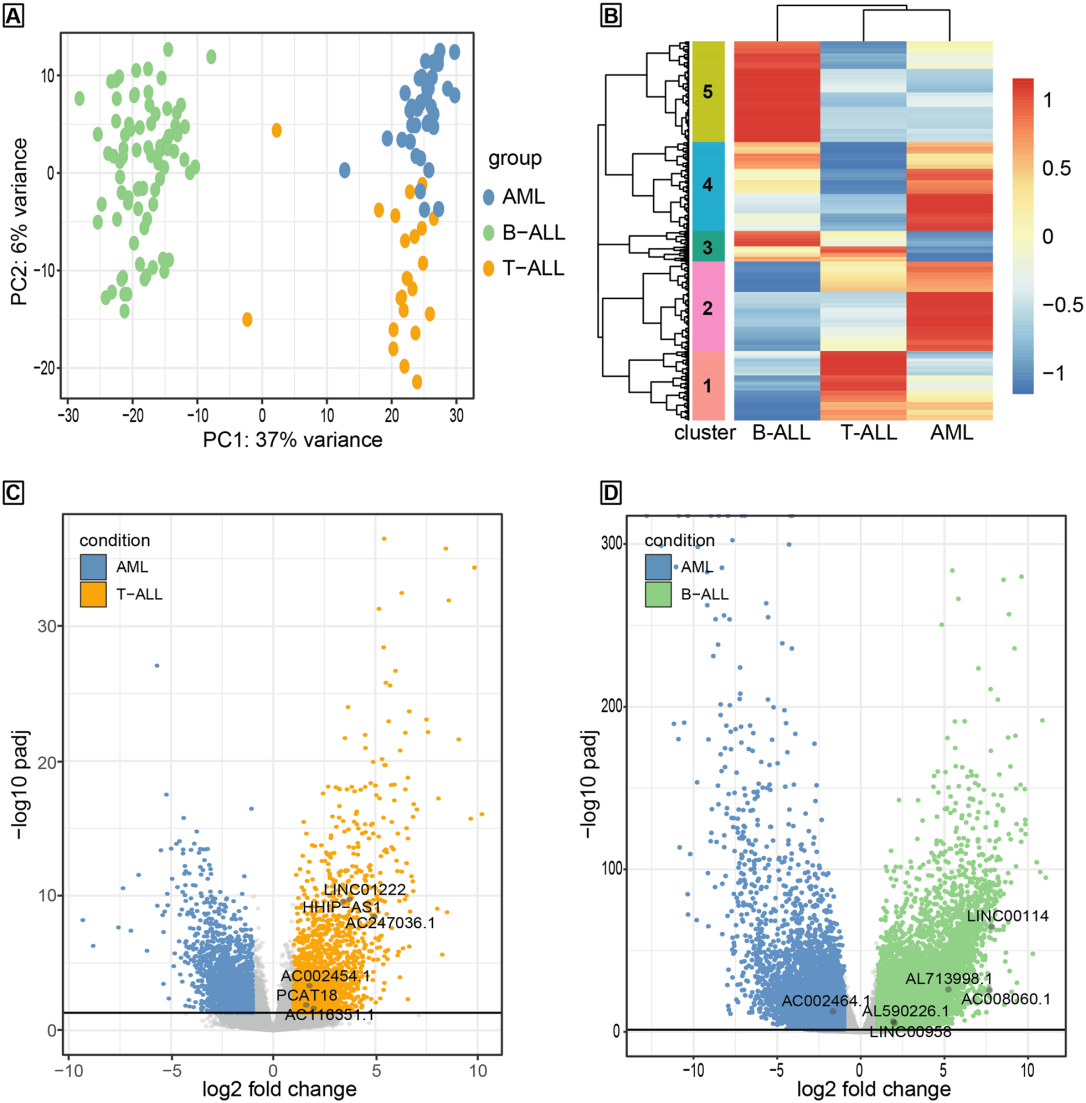
LncRNA landscape in paediatric ALL and AML. **A** PCA of the lncRNA landscape of childhood B-ALL, T-ALL and AML RNA-seq samples from Paediatric Cancer Genome Project by Saint Jude Children’s Research Hospital. **B** Hierarchical clustering output shows lncRNA expression in paediatric B-ALL, T-ALL and AML. Expression values, normalized by row, are indicated with a red to blue graded color. Each cluster comprises the main group of lncRNAs sharing a similar expression profile across the various diseases considered. **C** Volcano plot illustrating the transcriptome variations between AML and T-ALL. Significance and fold change threshold are reported as in Fig. 2B. Grey dots mark the position of the lncRNAs highlighted in Fig. 2C. **D** Volcano plot showing the transcriptomic differences between AML and B-ALL. Significance and fold change threshold are reported as in Fig. 2B. Grey dots mark the position of the lncRNAs highlighted in Fig. 2B

To increase our statistical power, we tested our findings in a larger cohort of childhood leukaemia patients using RNA-seq data from Paediatric Cancer Genome Project by Saint Jude Children’s Research Hospital (https://platform.stjude.cloud/data/cohorts/pediatric-cancer) [31]. We compared 80 B-ALL, 25 T-ALL, and 38 acute myeloid leukaemia (AML) samples. Also, in this case, the PCA analysis of lncRNAs’ panorama shows clear segregation of the different conditions, suggesting that the lncRNA signature could help distinguish among the three types of childhood leukaemia disease and not only between B-ALL and T-ALL. Surprisingly, the lncRNA signature of T-ALL patients appears to be more similar to the signature of AML patients than that of patients affected by the other type of acute lymphoblastic leukaemia examined, B-ALL (Fig. 4A). This data was confirmed by the hierarchical clustering of all the lncRNAs in B-ALL, T-ALL, and AML, where the dendrogram of the three conditions shows T-ALL and AML separating from B-ALL (Fig. 4B). The candidate lncRNAs individuated as T-ALL biomarkers from our RNA-seq analysis and following real-time qPCR validations all belong to cluster 1, except for HHIP-AS1 belonging to cluster 3 (complete list of clusters in Additional file 7: Dataset S7). However, this data is not in contrast with our previous findings, but on the contrary, it highlights the low expression of HHIP-AS1 in AML. The upregulation of HHIP-AS1 in T-ALL compared to AML is confirmed by the differential expression analysis pointing out a significant and consistent upregulation of all the T-ALL lncRNA biomarker candidates (Fig. 4C). Further, all the lncRNAs previously selected as T-ALL biomarkers were part of cluster 5, except for AC002464.1, which belongs to cluster 2. However, this lncRNA was unable to discriminate between B-ALL and T-ALL (Fig. 2D), qualifying as a general biomarker of leukemic transformation compared to the healthy control, not specific for any of the examined paediatric leukemias. Indeed, subsequent differential expression analysis showed that AC002464.1 is even upregulated in AML. In contrast, all the other B-ALL selected lncRNAs are significantly upregulated in B-ALL, with log2(Fold Change) always greater than 1.

### Gene expression correlation of T-ALL candidate lncRNAs

**Fig. 5:**
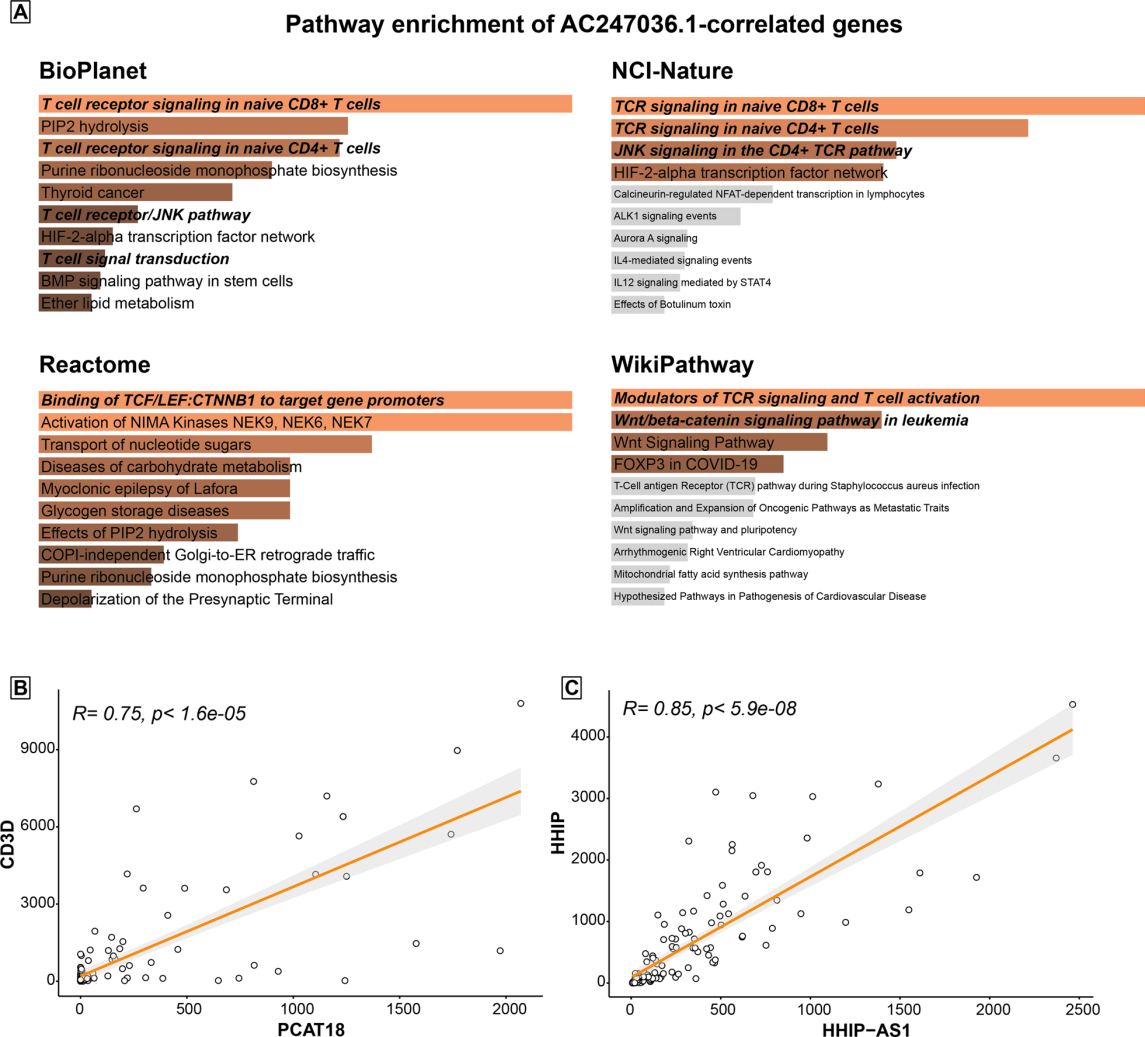
Correlation study of selected lncRNAs from paediatric T-ALL signature. **A** Pathway enrichment analysis results for all the genes correlated with AC247036.1 with a p-value < 0.001. The figure shows the results from different databases: BioPlanet [48], NCI-Nature [49], Reactome [50] and WikiPathway [29]. Terms are presented in crescent order of p-value (top down). Orange coloured terms are significant terms (p-value < 0.05), grey terms are not significant. Bold italic font highlights interesting enriched terms. **B** Expression correlation between PCAT18 and CD3D. **C** Expression correlation between HHIP-AS1 and HHIP

We focused our further bioinformatic analysis on the lncRNAs selected from T-ALL signature, due to both the particular aggressiveness of this disease and its rarity in paediatric age, which makes it even more difficult and scarcely feasible to study the molecular dynamics underlying its physiopathological mechanisms. For each T-ALL candidate lncRNAs individuated by our bioinformatics analyses, we calculated expression correlation with any other gene using all the Paediatric Cancer Genome Project samples by Saint Jude Children’s Research Hospital. This test highlighted some interesting correlations between the expression of the lncRNAs, ALL marker genes, and important players in the oncogenesis process (complete lists of correlated genes in Additional file 8: Dataset S8). For instance, the pathway enrichment analysis for all the genes positively correlated with AC247036.1 with a p-value < 0.001 returned significant enrichment for several terms regarding TCR signalling, T cell activation and β-catenin signalling pathway (Fig. 5A). Further, PCAT18, part of the T-ALL signature, is shown to be highly significantly correlated with the expression of the delta subunit of CD3 (CD3D), a well-known marker of T cell lineage [32] (Fig. 5B), confirming the specificity of this lncRNA to discriminate malignancy of T cells from acute leukemias of other blood cell types. Interestingly, we found HHIP-AS1 to be positively correlated with its sense adjacent coding transcript, HHIP, an inhibiting factor of Hedgehog signalling [33] (Fig. 5C). This correlation could suggest an implication of this lncRNA in the Sonic Hedgehog (SHH) pathway, an important proliferative factor that can underlying T-ALL oncogenic transformation [34]. This result is in agreement with a previous study showing HHIP-AS1 to be an important regulator of SHH pathway in solid tumours [35], and suggests an analogous HHIP-AS1 role also in leukemogenesis.

## Discussion

Nowadays, thanks to improvements in diagnostics and treatment protocols, the outcome for paediatric patients with acute leukaemia is quite favourable. Especially in the case of B-ALL, about 80% of children go through a full recovery. However, there are cases of relapses in which standard therapies are ineffective, leading to a poor prognosis. On the other hand, paediatric T-cell leukaemias often have a poorer prognosis due to their aggressiveness and resistance to many standard treatments [36]. It is indeed crucial to identify novel targets of the paediatric leukaemia to allow an accurate and timely choice of the treatment protocol most appropriate for the patient's clinical situation. This could be a tricky decision to make since childhood leukaemias are heterogeneous diseases. The advent of NGS has strongly boosted the identification of new biomarkers for use in diagnosis and/or therapy. Yet, these advances have been rapid but uneven. While some aspects have been studied in detail, such as cell surface protein and protein-coding genes that could be targeted in therapy protocols), other aspects have been overlooked, such as the molecular non-coding footprint underlying the disease. Our work aims to contribute to bridge this gap and finely characterise the lncRNA landscape of paediatric acute leukaemias. LncRNAs are a class of biomarkers of crescent interest in the haematologic and oncologic field [19, 29–31]. They do not encode proteins and have been reported by several studies to modulate gene expression at the transcriptional, post-transcriptional, and epigenetic levels [20]. In particular, due to their involvement in vital oncogenic processes such as differentiation, proliferation, migration, angiogenesis, and apoptosis, lncRNAs have attracted much attention as potential diagnostic and prognostic biomarkers in leukaemia [40, 41].

Starting from NGS transcriptome analyses of B-ALL and T-ALL patients in comparison with B and T lymphocytes from cord blood, we identified a specific lncRNAs signature able to discriminate B-ALL and T-ALL not only from healthy subjects but also between the two types of leukaemia. We selected some candidate lncRNAs that have never been associated with ALL and tested their expression in a larger cohort of patients. For most of them, this experiment confirmed the expression absence in the healthy patient and a significant upregulation in a specific type of ALL, hinting at a potential diagnostic application in clinical practice. Further, we found a significant negative correlation of AC247036.1 with WBC at diagnosis, that is historically considered a risk factor for treatment failure [42–44]. This data highlighted AC247036.1 as a possible favourable prognostic factor for T-ALL treatment success.

Furthermore, we showed that the lncRNA landscape is specific not only for the two paediatric lymphoblastic leukaemias (B-ALL and T-ALL) but also for myeloid ones. Interestingly, the T-ALL lncRNA signature is somewhat more related to AML than B-ALL, despite the great etiopathological difference between the two diseases. This finding was unexpected. However, it is important to consider that both T-ALL and AML may have common traits in the case of leukemic transformation. Specifically, in the case of AML transformation, it is more likely to find the ectopic expression of T-cell-associated antigens ( such as CD2, CD5 and CD7) than B-cell ones (CD19, CD20) [45]. In particular, the CD7 antigen was found to be expressed in 30% of de novo AML and some authors proposed to use the ectopic expression of this antigen for planning AML blasts specific CAR-T therapy Identifying common traits between AML and T-ALL in terms of lncRNA could open a novel scenario to investigate on altered pathways leading to leukemogenesis and characteristics of aggressiveness [46]. Our data showed a certain similarity between these two diseases also for the lncRNA landscape. To further investigate the issue, we discriminated between high CD7-content-AML and low CD7-content-AML. Highlighting these two types of AMLs in the PCA analysis with T-ALL and B-ALL, we found that even if the spatial distribution is still heterogeneous, high CD7-content-AML patients are the closest with T-ALL patients, sometimes even intermingling in the same cluster (Additional file 11: Fig. S3).

In the final part of this work, since the role of many lncRNAs involved in childhood acute lymphoblastic leukaemia is still unknown, especially in T-ALL, we performed correlation analyses to try to identify the potential role of the lncRNAs in this pathology. Our in silico analyses revealed a gene ontology enrichment in the key pathways for T-cell differentiation for those genes positively correlating with AC247036.1, suggesting its potential role in the modulation of some genes involved in these processes associated with leukemogenesis. Further, we found that lncRNA PCAT18 was associated with the expression of CD3D antigen, a well-known T-cell marker used in diagnostics for monitoring minimal residual disease by flow cytometry. This finding is new and confirmed the relationship between the PCAT18 lncRNA expression and T cell lineage commitment, however additional functional experiments are needed to evaluate the role of PCAT18 in sustaining leukemic growth. Last, our data disclose a highly significant positive correlation between the lncRNA HHIP-AS1 and its relative sense protein-coding transcript HHIP [33]. This probably happens because HHIP-AS1 is actively transcribed from a SHH-responsive bidirectional promoter shared with the SHH signalling intermediate HHIP. In SHH-driven tumours, the knockdown of HHIP-AS1 induces mitotic spindle deregulation and the consequential reduction of tumorigenicity in vitro and in vivo [47]*.* Taken together, these data suggest HHIP-AS1 to be a suitable candidate for further functional studies to explore its possible role in enabling the pro-mitotic effects of SHH pathway activation in childhood T-ALL.

Ultimately, our work made available to the research community a comprehensive map of the lncRNA landscape of the various types of paediatric leukaemia, useful not only for diagnostic purposes but also, after appropriate ad hoc functional studies, for therapeutic purposes. However, it is important to remark the pilot nature of this study due to the reduced sample size. Further studies with a larger cohort of patients will be needed to consistently correlate the expression levels of target lncRNAs to patients' clinical information in order to disclose a possible lncRNA prognostic role contributing to risk stratification and, therefore, to an improvement in the clinical management of the paediatric patients. In this respect, it is important to note that lncRNAs are easily detectable. Their identification could be included in normal clinical practice strengthening the diagnostic process and improving paediatric patient management. Increasing the patient cohort could help to correlate the expression of specific candidate lncRNAs identified by our study with clinical information, testing their potential prognostic effectiveness in stratifying patients according to their clinical characteristics. This aspect made our study an important resource for the scientific community, laying the foundations for future functional and clinical studies.

## Conclusion

In conclusion, here we presented an extended analysis of the lncRNA profile for B-ALL, T-ALL as well as cord blood-derived T and B cells. Specific lncRNA signatures were detectable in the case of B-ALL and T-ALL. In the case of T-ALL it was interesting to find that PCAT18 was strongly associated with the expression of the CD3D, a T cell lineage specific antigen. Moreover, HHIP-AS1 appeared to be associated with the SHH pathway, that is frequently deregulated in T-ALL. Although the observational nature of our study, it made available to the research community a comprehensive map of the lncRNA landscape of the various types of paediatric leukaemia, useful not only for diagnostics purposes but also, after appropriate ad hoc functional studies, for therapeutic purposes. In this respect, it is important to note that lncRNAs are easily detectable. Their identification could be included in normal clinical practice strengthening the diagnostic process and improving paediatric patient management. Increasing the patient cohort could help to correlate the expression of specific candidate lncRNAs identified by our study with clinical information and testing their potential prognostic effectiveness in stratifying patients according to their clinical characteristics. This aspect made our study an important resource for the scientific community, laying the foundations for future functional and clinical studies.

## Supplementary Information

**Additional file 1: Dataset S1.** B-ALL patients clinical information.

**Additional file 2: Dataset S2.** T-ALL patients clinical information.

**Additional file 3: Dataset S3.** T-ALL differential gene expression analysis.

**Additional file 4: Dataset S4.** B-ALL differential gene expression analysis.

**Additional file 5: Dataset S5.** Complete list of lncRNA molecular signature.

**Additional file 6: Dataset S6.** Complete differential analysis of lncRNAs in B-ALL VS T-ALL.

**Additional file 7:** **Dataset S7.** Complete gene list of clusters.

**Additional file 8:** **Dataset S8.** Complete lists of lncRNAs correlated genes.

**Additional file 9:** **Dataset S9.** Counts of RNA-seq sequencing samples of B-ALL patients.

**Additional file 10:** **Dataset S10.** Counts of RNA-seq sequencing samples of T-ALL patients.

**Additional file 11: Figure S1.** Whole and protein coding transcriptome in paediatric ALL. **A **Number of common upregulated transcripts between the comparison T-ALL vs T naïve cells and B-ALL vs B naïve cells. Fisher’s exact test scored the overlapping as significant with a p-value = 1.1^e-56^. **B **Number of common upregulated protein coding RNAs between the comparison T-ALL vs T naïve cells and B-ALL vs B naïve cells. Fisher’s exact test scored the overlapping as significant with a p-value = 5.4^e-58^. **C **PCA of the whole transcriptome of all the samples. **D **PCA of the protein coding transcriptome of all the samples. **Figure S2.** Correlation between lncRNA expression and clinical information. **A **Pearson correlation between AC247036.1 expression level in T-ALL patients from RT-PCR and WBC at diagnosis. **B **Pearson correlation between HHIP-AS1 expression level in T-ALL patients from RT-PCR and patients’ age. **Figure S3.** LncRNA landscape in paediatric ALL and AML. PCA of the lncRNA landscape of childhood B-ALL, T-ALL and AML RNA-seq samples from Paediatric Cancer Genome Project by Saint Jude Children’s Research Hospital. Different shades of blue differentiate between low CD7-content-AML and high CD7-content-AML. The threshold to discriminate high-CD7-content AMLs and low-CD7content AMLs was set based on the median of normalized RNA-seq counts of expression.

## Data Availability

Counts of RNA-seq sequencing samples are available in Additional file 9: Dataset S9 (B-ALL raw counts) and Additional file 10: Dataset S10 (T-ALL raw counts). Raw sequences are from under-aged human patients, hence FASTQ files will be available upon request. Contact corresponding author G. S. (giovanni.smaldone@synlab.it).

## Abbreviations

ALL: Acute lymphoblastic leukemia
B-ALL: Acute lymphoblastic leukemia of B-cells
lncRNA: Long non coding RNA
NGS: Next generation sequencing
PBMC: Peripheral blood mononuclear cells
T-ALL: Acute lymphoblastic leukemia of T-cells
