# Supplementary material for: Specific lncRNA signatures discriminate childhood acute leukaemias: a pilot study
Source: Cancer Cell Int. 2022 Nov 30;22:373. doi: 10.1186/s12935-022-02789-3 (PMC9710039; doi:10.1186/s12935-022-02789-3)
Supplement: Supplementary file 11 — Additional file 11: Figure S1. Whole and protein coding transcriptome in paediatric ALL. A Number of common upregulated transcripts between the comparison T-ALL vs T naïve cells and B-ALL vs B naïve cells. Fisher’s exact test scored the overlapping as significant with a p-value = 1.1e-56. B Number of common upregulated protein coding RNAs between the comparison T-ALL vs T naïve cells and B-ALL vs B naïve cells. Fisher’s exact test scored the overlapping as significant with a p-value = 5.4e-58. C PCA of the whole transcriptome of all the samples. D PCA of the protein coding transcriptome of all the samples. Figure S2. Correlation between lncRNA expression and clinical information. A Pearson correlation between AC247036.1 expression level in T-ALL patients from RT-PCR and WBC at diagnosis. B Pearson correlation between HHIP-AS1 expression level in T-ALL patients from RT-PCR and patients’ age. Figure S3. LncRNA landscape in paediatric ALL and AML. PCA of the lncRNA landscape of childhood B-ALL, T-ALL and AML RNA-seq samples from Paediatric Cancer Genome Project by Saint Jude Children’s Research Hospital. Different shades of blue differentiate between low CD7-content-AML and high CD7-content-AML. The threshold to discriminate high-CD7-content AMLs and low-CD7content AMLs was set based on the median of normalized RNA-seq counts of expression. [file 12935_2022_2789_MOESM11_ESM.pdf]

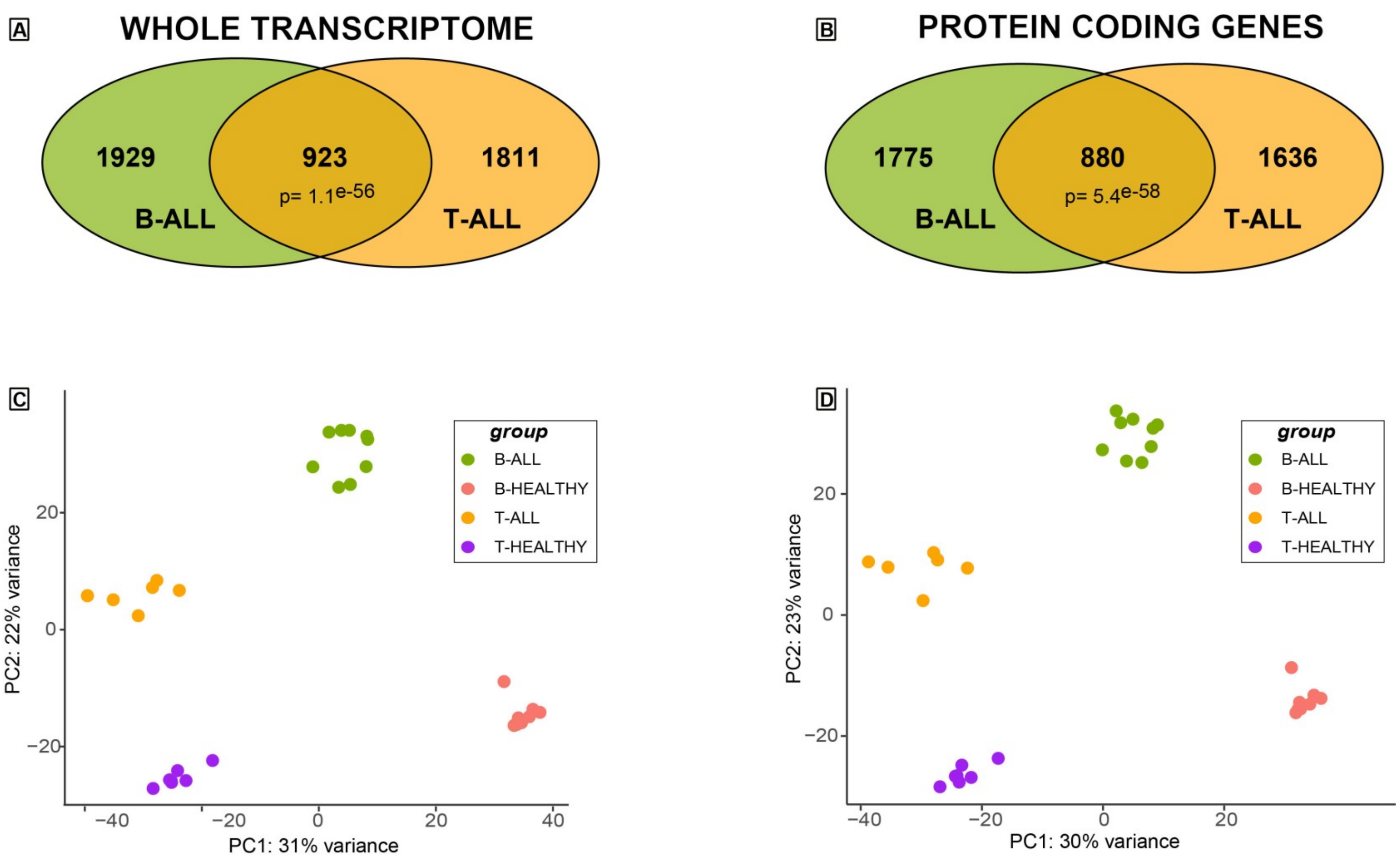

Figure S1. ***Whole and protein coding transcriptome in paediatric ALL***. **A** Number of common upregulated transcripts between the comparison T-ALL vs T naïve cells and B-ALL vs B naïve cells. Fisher's exact test scored the overlapping as significant with a p-value = 1.1^{e-56}. **B** Number of common upregulated protein coding RNAs between the comparison T-ALL vs T naïve cells and B-ALL vs B naïve cells. Fisher's exact test scored the overlapping as significant with a p-value = 5.4^{e-58}. **C** PCA of the whole transcriptome of all the samples. **D** PCA of the protein coding transcriptome of all the samples.

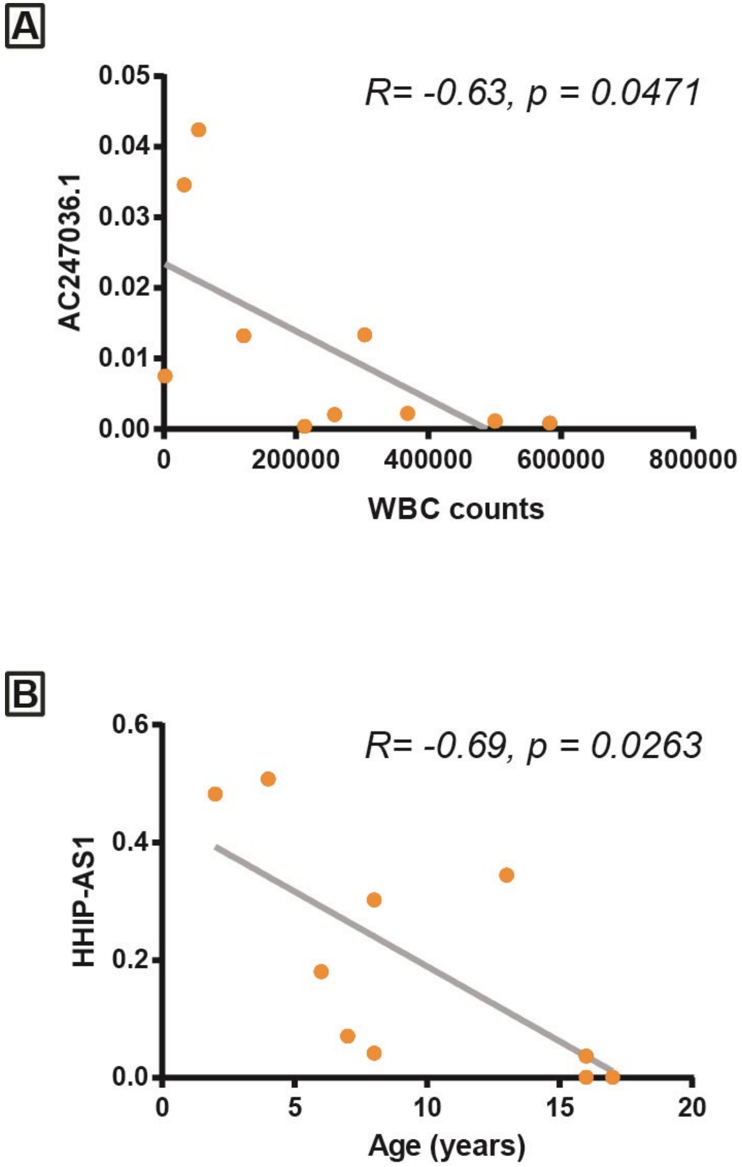

Figure S2. ***Correlation between lncRNA expression and clinical information***. **A** Pearson correlation between AC247036.1 expression level in T-ALL patients from RT-PCR and WBC at diagnosis. **B** Pearson correlation between HHIP-AS1 expression level in T-ALL patients from RT-PCR and patients' age.

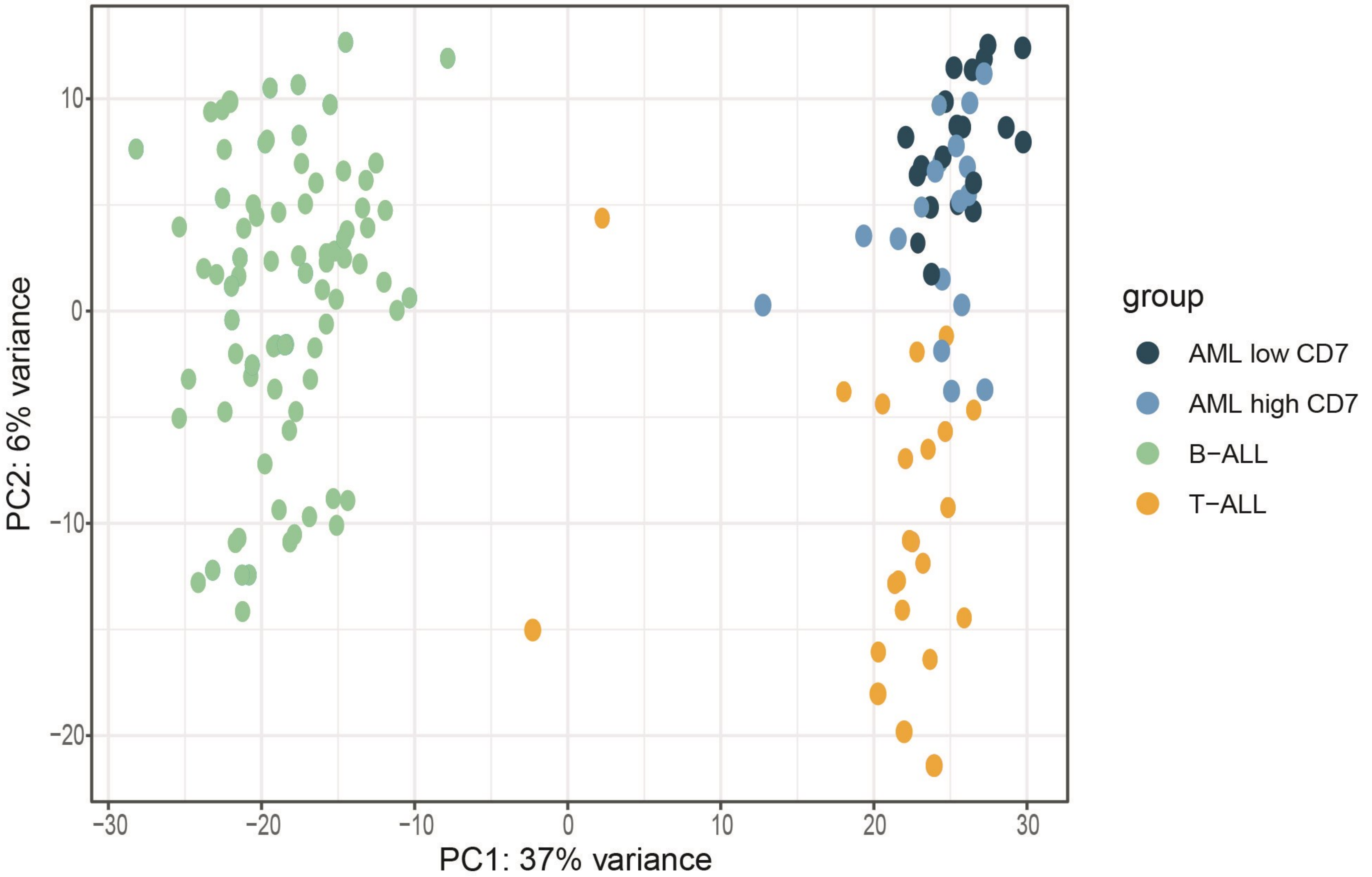

Figure S3. ***LncRNA landscape in paediatric ALL and AML***. PCA of the lncRNA landscape of childhood B-ALL, T-ALL and AML RNA-seq samples from Paediatric Cancer Genome Project by Saint Jude Children's Research Hospital. Different shades of blue differentiate between low CD7-content-AML and high CD7-content-AML. The threshold to discriminate high-CD7-content AMLs and low-CD7-content AMLs was set based on the median of normalized RNA-seq counts of expression.
